# Efficacy analysis of rituximab in treating patients with primary membranous nephropathy dependent on calcineurin inhibitors

**DOI:** 10.3389/fimmu.2024.1504646

**Published:** 2024-12-18

**Authors:** Zhuo Li, Tingting Zhao, Shasha Zhang, Jing Huang, Honggang Wang, Yujiao Sun, Rong Wang, Bing Chen

**Affiliations:** ^1^ Department of Nephrology, Shandong Provincial Hospital Affiliated to Shandong First Medical University, Jinan, Shandong, China; ^2^ Department of Respiratory and Critical Care Medicine, Shandong Public Health Clinical Center, Shandong University, Jinan, Shandong, China; ^3^ Department of Nephrology, Shanghai East Hospital, Tongji University School of Medicine, Shanghai, China; ^4^ Department of Nephrology, Jinan Shizhong People’s Hospital, Jinan, China; ^5^ Department of Nephrology, The Second Hospital of Shandong University, Jinan, Shandong, China

**Keywords:** primary membranous nephropathy, rituximab, withdrawal drug rate, clinical remission rate, immunologic remission rate

## Abstract

**Background:**

This study evaluated the efficacy of rituximab (RTX) in primary membranous nephropathy (PMN) patients with incomplete remission and drug dependence after long-term use of calmodulin inhibitors (CNIs). It aims for complete clinical and immunological remission, and cessation of CNI dependence.

**Methods:**

Thirty-six patients were enrolled in the study with two groups: drug-dependent and partial remission or immune non-remission group. Both groups underwent RTX therapy with gradual CNI tapering to end CNI dependency and induce complete remission. The primary outcome was overcoming CNI dependency and achieving complete remission after 12 months of RTX therapy. Secondary outcomes included immunological remission and recurrence rates.

**Results:**

The drug-dependent group (20 patients) achieved significant proteinuria reduction compared to the partial remission or immune non-remission group (16 patients) (*P*=0.016). After 12 months of RTX treatment, all drug-dependent patients overcame CNI dependency (average withdrawal period: 5.3 ± 3.7 months), with complete remission rates increased from 10% to 70.0% and complete immunological remission rates rose from 35.0% to 90.0%. In the partial remission or immune non-remission group, 14 patients discontinued CNI (average period: 4.6 ± 4.5 months), with complete remission rates increasing from 5.0% to 68.8% and complete immunological remission rates from 6.3% to 68.8%. During follow-up, serum albumin increased, and anti-PLA2R antibodies, 24-hour proteinuria, and CD19^+^ cell numbers reduced, while creatinine remained stable. Three patients relapsed, four encountered adverse events, and no malignancies or other fatal adverse events were reported.

**Conclusions:**

RTX effectively achieves complete clinical and immunological remission in PMN patients dependent on or partially responsive to long-term CNI therapy, reducing recurrence and minimizing prolonged immunosuppressive therapy risks.

## Introduction

Primary membranous nephropathy (PMN) is the most common cause of nephrotic syndrome (NS) in adults and is characterized as an autoimmune disease targeting podocytes. It involves the formation of subepithelial immune deposits, primarily immunoglobulin G (IgG), and complement activation, mainly complement protein 3 (C3), which contribute to podocyte dysfunction and the onset of NS (1, 2). PLA2R remains the optimal marker for diagnosing, risk stratification, predicting the likelihood of spontaneous remission, and evaluating the response to immunosuppressive therapy in primary MN, being identified in 74-78% of the cases of pMN (3, 4).The identification of these antigens has justified the use of B-cell depleting agents, as B-cell depletion and reduction of anti-PLA2R antibody lead to the remission of proteinuria (5–7), highlighting the crucial role of anti-PLA2R antibody levels in predicting clinical outcomes, evaluating therapeutic responses, and guiding immunosuppressive therapy (6, 8).

Current first-line regimens for PMN include glucocorticoids (GC) combined with either alkylating agents (cyclophosphamide, CYC, or chlorambucil) or calcineurin inhibitors (CNIs, such as cyclosporin A, CsA, or tacrolimus, TAC) (9, 10). These medications, however, lack specificity and present significant side effects. For example, CYC can induce severe toxicities, including myelosuppression, infection, gonadal suppression, increased cancer risk (11), while CNIs are associated with chronic nephrotoxicity and a high recurrence rate (40%–50%) (12), often leading to drug dependency (11, 13, 14). Our previous research (15)compared the effects of CYC and CNI in treating PMN, demonstrating drug dependency in patients treated with both. Specifically, patients receiving CNI were less likely to successfully discontinue treatment compared with those receiving CYC (28% vs. 76.36%, *P*< 0.001). Clinically, a significant number of patients develop CNI dependency, increasing their long-term reliance on immunotherapy and risk of nephrotoxicity. Abrupt discontinuation can lead to high recurrence rates, underlining the need for new strategies to address the current treatment dilemma.

Rituximab (RTX) is a human–mouse chimeric monoclonal antibody specifically directed against the B-cell surface antigen CD20. It depletes CD20^+^ B cells through mechanisms such as antibody-dependent cellular cytotoxicity, complement-dependent cytotoxicity, and direct induction of apoptosis. CD20 antigen, expressed on the surface of early and mature B lymphocytes but not on hematopoietic stem cells, normal plasma cells, or other normal tissues, allows RTX to selectively reduce B lymphocyte populations and inhibit autoantibodies production without nonspecific immunosuppression (16).The 2021 KDIGO guidelines recommend RTX for the treatment of PMN, with its safety and efficacy well-documented in multiple studies (17–20).

Despite its benefits, previous research has reported limited patient demographics inRTX trials. The MENTOR trial (18), for instance, compared RTX’s efficacy with CsA, focusing on patients new to immunotherapy, while excluding those previously treated with immunosuppressives. Zhao Ming-Hui et al. (21) reported RTX’s efficacy in refractory PMN patients, including those in a persistently unremitted state. Our study also examined another clinical subset: patients who achieved clinical remission with CNI treatment but developed dependency, with proteinuria re-emerging upon dosage reduction or discontinuation. These patients were given RTX with a subsequent gradual reduction in CNI, allowing researchers to evaluate RTX’s role in maintaining clinical remission and managing drug dependency. Patients who received long-term (>12 months) standard-dose CNI and achieved only partial or no immunological remission were also included, noting a high recurrence rate and necessitating ongoing RTX treatment while tapering off CNI.

This study employed a comprehensive approach to assess patient conditions by monitoring prognostic biomarkers, such as proteinuria levels, anti-PLA2R antibody titers, and B-cell counts. This monitoring helped determine the optimal timing for RTX administration while managing the reduction rate of CNI/GC, facilitating their gradual discontinuation.

## Materials and methods

### Patient population

This study included 42 adults (>18 years old) with PMN treated at the Nephrology Department of Shandong Provincial Hospital from June 2019 to June 2022. After excluding two cases because of irregular RTX dosing and four cases with insufficient follow-up duration (<12 months), 36 patients were retrospectively enrolled. All were diagnosed with PMN via renal biopsy. Patients were categorized into two groups (1): a drug-dependent group, which achieved complete remission with standard-dose CNI treatment but experienced an increase in proteinuria (>0.5 g/24 h)following CNI tapering—remission was reinstated with the re-administration of CNIs, indicating the necessity of ongoing medication; and (2) a partial remission or immune non-remission group, which only achieved partial remission or ceased proteinuria without full immunological remission after long-term (>12 months) standard-dose CNI therapy. Both groups received RTX treatment with gradual CNI cessation based on their clinical responses.

Comprehensive clinical and laboratory records were maintained for all patients. Through medical histories, physical examinations, and laboratory tests (including serology and imaging), causes of secondary membranous nephropathy, such as underlying malignancies, pathogenic drugs, hepatitis B/C viruses, HIV, and autoimmune diseases were ruled out.

This study was carried out in accordance with the Helsinki Declaration, and the study protocol was approved by the Ethics Review Committee of Shandong Provincial Hospital in China (JNKJ: NO. 2020-3028).

### Treatment program and follow-Up

Two RTX dosing regimens were employed: (1) RTX administered intravenously at 375 mg/m^2^ once a week for 3–4 doses, or (2) RTX administered intravenously at 1 g/dose for 1–2 doses at 2-week intervals. The 2021 KDIGO guidelines recommend both regimens for PMN patients (9), citing studies that show no significant differences in the rates of complete response (CR) or partial response (PR), or in side effects between the regimens (22, 23). Six months post-treatment, the decision to repeat the injection of RTX was based on the patient’s medication reduction, B-cell rebound, anti-PLA2R antibody levels, and clinical remission status; the assessment of whether to repeat the injection was repeated approximately every 6 months thereafter. RTX was dissolved in 9% saline to a concentration of 1 mg/mL and infused at an initial rate of 40 mL/h, then gradually increased to 200 mL/h as tolerated by each patient. To reduce infusion reactions to RTX, patients received 40 mg methylprednisolone, 5 mg dexamethasone, and 25 mg iproniazid before injection.

Standard CNI dosing regimens included: for TAC, an initial oral dose of 0.05–0.1 mg/(kg•d),adjusted based on plasma levels measured 1–2 weeks later to maintain a target range of 5–10 ng/mL for at least 12 months; for CsA, an initial dose of 3–5 mg/(kg•d) was adjusted based on drug levels to maintain 100–200 ng/mL, and the treatment was continued for at least 12 months.

Tapering regimen included: (1) After regular RTX therapy, the GC dose was reduced by one tablet every 2 weeks to a minimum of one tablet for maintenance and tapered off within 2–3 months; (2) Depending on patient condition, the CNI dose was reduced to TAC 1 mg twice daily or CsA 50 mg twice daily, maintained for 2–3 months, and then reduced by one tablet per month until withdrawal.

Patients were followed up every 3 months, which includes assessments before RTX treatment and at the 3rd, 6th, 9th, and 12th months post-treatment. Each follow-up included routine blood tests, urine tests, liver and kidney function tests, lipid profiles, blood glucose, 24-hour urine protein quantification, anti-PLA2R antibody levels, and circulating B-cell counts. Anti-PLA2R antibody titers were measured using a standardized commercial ELISA method (Euroimmune, Lubeck, Germany), with titers <20 U/mL defined as negative and <2 U/mL as complete immunological remission. B-cell depletion was defined as a concentration of<5 B cells/mm^3^ in the circulation. Patient medication reduction and remission status were recorded, along with adverse events related to RTX during infusion and throughout the follow-up period. Subsequent follow-ups were conducted every 6 months to document remission and relapse.

### Efficacy assessment and renal outcomes

The primary endpoint was to assess the overcoming of CNI dependency and the achievement of complete remission by the 12th month post-RTX treatment. Secondary endpoints included achieving complete immunological remission and evaluating patient relapse rates. CR was defined as urinary protein <0.3g/24h with stable renal function (estimated glomerular filtration rate, eGFR ≥45 mL/min/1.73 m^2^). PR was defined as urinary protein levels between 0.3–3.5 g/24 h with either a >50% reduction from baseline or maintenance of serum albumin levels >30g/L, provided renal function remains stable (eGFR ≥45 mL/min/1.73 m^2^). Patients failing to meet these criteria were categorized as non-responders who did not achieve clinical remission. Immunological remission was defined as an anti-PLA2R antibody titer <2 U/mL. Complete relapse was defined as the recurrence of urinary protein levels>3.5 g/24 h following remission, whereas a partial relapse was indicated by a level above 0.5g/24h. Overcoming drug dependence was defined as complete cessation of CNI therapy while in clinical remission. The primary observational endpoint for renal outcomes included the deterioration of renal status or progression to end stage renal disease (ESRD). A deterioration in renal status was defined as a post-treatment rise in serum creatinine of >133 µmol/L or a doubling of baseline serum creatinine levels lasting more than 3 months. ESRD was defined as a creatinine clearance of <15 mL/min at the last follow-up, initiation of hemodialysis, or at the time of renal transplantation. Serious adverse events were defined as either clinical death, serious pulmonary infection, pulmonary embolism, cerebral infarction, myocardial infarction, or hospitalization due to adverse events.

### Statistical analysis

SPSS 22.0 was used for statistical analyses. Continuous variables with a normal distribution were expressed as mean ± standard deviation, with intergroup differences assessed using the *t*-test. Non-normally distributed variables were expressed as median (interquartile range)and compared using the Mann−Whitney U test or the Kruskal−Wallis test. Categorical variables were expressed as frequencies, and χ^2^ tests were used for all intergroup comparisons. All tests were two-tailed, and statistical significance level was defined as *P*< 0.05,with*P <* 0.01 indicating high significance.

## Results

### Baseline data

**Figure 1:**
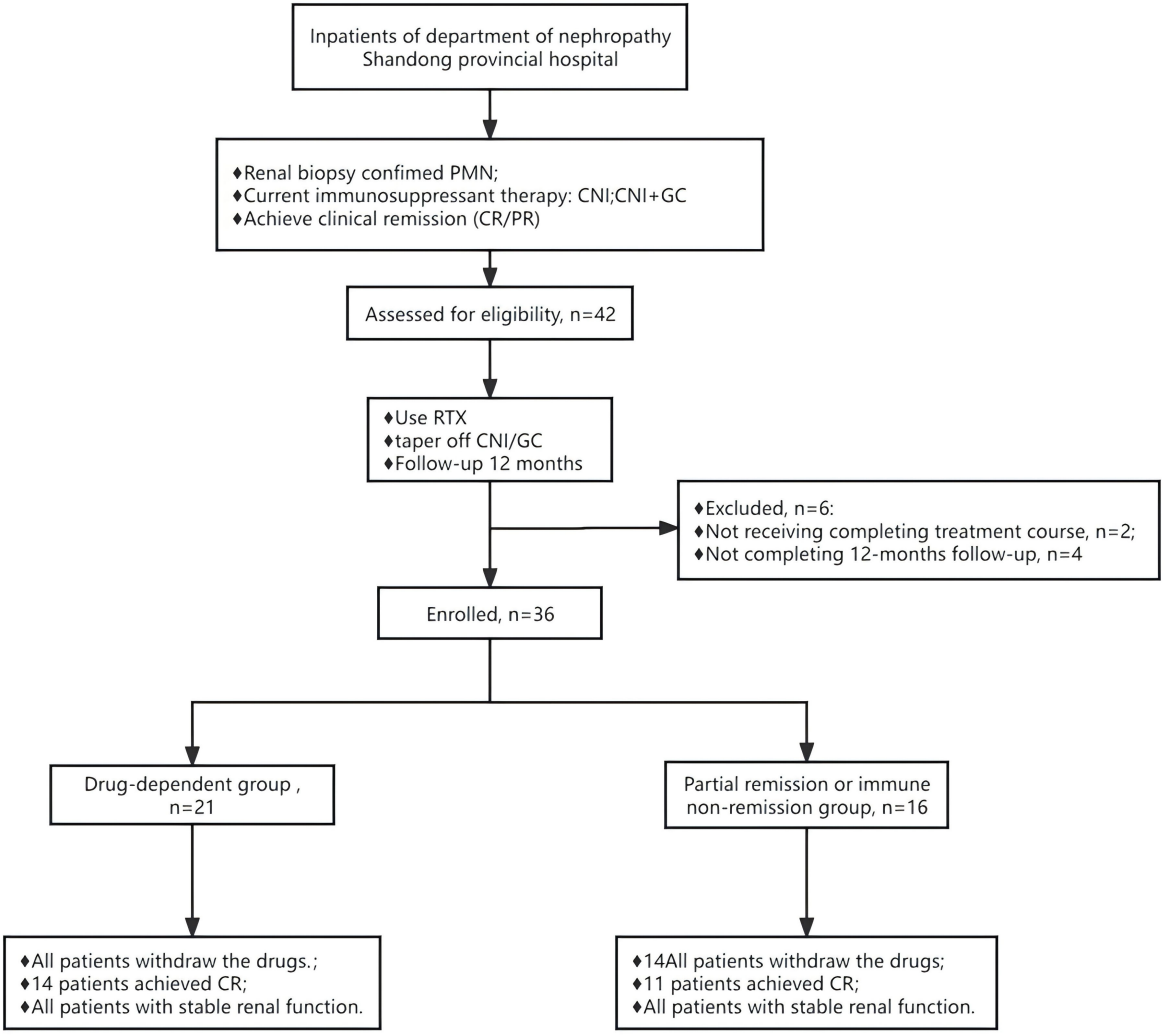
Flow chart of the patients with primary membranous nephropathy receiving rituximab therapy. GC+CNI, glucocorticoids combined with calcineurin inhibitor. RTX, rituximab.

This study included 36 PMN patients, divided into two groups: 20 in the CNI-dependent group and 16 in the partial remission or immune non-remission group, as shown in **Figure 1**. Prior to receiving RTX treatment, the median urinary protein level was 1.3 (0.5, 2.4) g/24 h, with a mean serum albumin level at 35.5 ± 4.3 g/L, mean serum creatinine at 69.8 ± 16.1 µmol/L, and mean eGFR at 101.1 ± 24.4 mL/min/1.73m^2^. The average level of serum anti-PLA2R antibodies was 17.9 ± 29.6 U/mL, with eight patients testing positive (titer >20 U/mL), 18 with titers <4 U/mL, and 10 with titers <2 U/mL. Therefore, 26 patients (72.2%) did not achieve immunological remission (>2 U/mL), including 13 (65.0%) in the drug-dependent group and 13 (81.3%) in the partial remission or immune non-remission group. The level of urinary protein in the drug-dependent group was significantly lower than in the partial remission or immune non-remission group, with a significant difference between the groups (*P* = 0.016), as detailed in **Table 1**.

**Table 1:** Baseline characteristics of patients with PMN included in this study.

Characteristic	Total (n=40)	Drug-dependent group (n=21)	Partial remission or immune non-remission group (n=16)	*P*
Male sex, n (%)	15 (41.7)	8 (40.0)	7 (43.8)	0.821
Age (years)	44.9 ± 13.1	41.4 ± 14.1	48.7 ± 11.1	0.108
Proteinuria (g/24h)	1.3 (0.5, 2.4)	0.7 (0.5,1.6)	2.0 (1.0,2.7)	**0.015**
WBC (×109/L)	6.7 (5.7, 8.3)	6.8 (6.3, 8.3)	6.6 (5.1, 8.4)	0.417
Hemoglobin (g/L)	122.5 (114.0, 143.0)	128.0 (115.0, 147.5)	118.5 (106.5, 137.0)	0.176
Platelet (×109/L)	261.5 (232.8, 316.0)	263.0 (234.8, 319.3)	257.0 (222.3, 316.0)	0.691
AST (u/L)	19.7 ± 6.9	19.6 ± 7.4	19.9 ± 6.4	0.890
ALT (u/L)	19.5 ± 9.2	19.3 ± 10.1	19.8 ± 8.2	0.887
Total protein (g/L)	60.5 ± 7.4	62.1 ± 7.3	58.5 ± 7.3	0.144
Albumin (g/L)	35.4 ± 4.3	36.5 ± 5.1	34.0 ± 2.5	0.081
Globulin (g/L)	25.3 ± 3.7	24.9 ± 3.7	25.8 ± 3.7	0.453
BUN (mmol/L)	6.5 ± 2.5	6.2 ± 2.6	7.0 ± 2.4	0.350
Serum creatinine(μmol/L)	69.8 ± 16.1	70.2 ± 17.2	69.4 ± 15.1	0.891
eGFR (mL/min/1.73 m2) ^a^	101.1 ± 24.4	100.9 ± 23.7	102.0 ± 26.1	0.890
Cholesterol (mmol/L)	5.6 ± 1.3	5.6 ± 1.4	5.7 ± 1.2	0.825
Anti-PLA2R antibodies (U/mL)	4.2 (2.0, 16.4)	3.5 (2.0, 11.3)	5.6 (2.6, 20.5)	0.792
Anti-PLA2R antibody positivity, n (%) ^b^	8 (22.2)	4 (20.0)	4 (25.0)	0.720
Anti-PLA2R antibodies titers ≤2U/mL, n(%)	10 (27.8)	7 (35.0)	3 (18.8)	0.279
The absolute values of CD19(/μl)	259.5 (68.9, 334.2)	273.3 (68.9, 362.3)	220.6 (38.1, 309.1)	0.394

Patients in the drug-dependent group had lower proteinuria levels(*P*=0.015) than those in the combination therapy group.

Values are presented as numbers (%), medians (interquartile range), or means ± SD.

PMN, primary membranous nephropathy; RBC, red blood cell; WBC, white blood cell; AST, aspartate aminotransferase; ALT, alanine aminotransferase; BUN, blood urea nitrogen; eGFR, estimated glomerular filtration rate; PLA2R, phospholipase A2 receptor.

^a^eGFR was calculated according to the Chronic Kidney Disease Epidemiology Collaboration equation.

^b^Anti-PLA2R positivity was defined by a value >20 U/mL.

Values in bold represent *P*<0.05.

Twenty-one patients received the first dosing regimen of RTX (8 received 3 doses and 13 received 4 doses), while 15 patients were administered the second regimen (6 received 1 dose and 9 received 2 doses); 15 patients underwent a second induction treatment 6 months after completing the first course, as detailed in **Table 2**.

**Table 2:** Dosing schedule for patients on rituximab.

	Initial induction therapy (n=36)	Second induction treatment (n=25)
RTX 375mg/m², weekly, 1 time	0	16
RTX 375mg/m², weekly, 2 times	0	5
RTX 375mg/m², weekly, 3 times	8	0
RTX 375mg/m², weekly, 4 times	13	0
RTX 1g/dose, 2 weeks interval, 1 time	6	4
RTX 1g/dose, 2 weeks interval, 2 times	9	0

RTX, rituximab.

### Efficacy assessment

**Figure 2:**
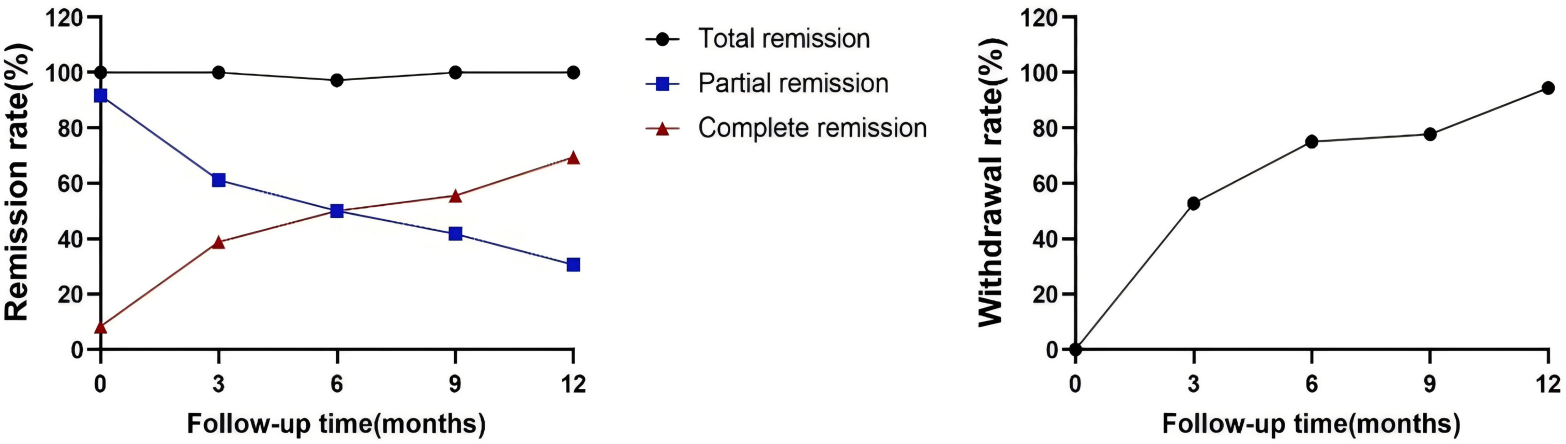
Changes in clinical remission rates. The primary outcome was the drug withdrawal rate and complete remission at 12 months. One patient was infected with a novel coronavirus in the fifth month after the first induction therapy and was subsequently hospitalized with pneumonia. During this period, he developed elevated proteinuria, and with the remission of the disease, the patient re-achieved clinical remission.

All patients completed a minimum of 12 months of follow-up, with all remaining in clinical remission by the 12th month. All patients in the drug-dependent group successfully overcame CNI dependency, with the average withdrawal time of 5.3 ± 3.7 months. None of these patients resumed any immunosuppressants post-discontinuation. The complete remission rate improved significantly, from 10% (2/20) to 70.0% (14/20), and the rate of complete immunological remission rose from 35.0% (7/20) to 90.0% (18/20). In the partial remission or immune non-remission group, 14 patients discontinued CNI, with the average withdrawal time of 4.6 ± 4.5 months). Here, the complete remission rate increased from 5.0% (1/20) to 68.8% (11/16), and the complete immunological remission rate increased from 6.3% (1/16) to 68.8% (11/16), as shown in **Table 3**. During the follow-up period, the cumulative rates of total CR and the rate of withdrawal from the drug increased over time, as illustrated in **Figure 2**.

**Table 3:** Complete remission and CNI withdrawal rate at 3–12 months.

Study Time Points	Total, n (%)		Drug-dependent group, n (%)		Partial remission or immune non-remission group, n (%)	
	Complete remission	Withdrawal rate	Complete remission	Withdrawal rate	Complete remission	Withdrawal rate
Before treatment	3 (8.3)	–	2 (10.0)	–	1 (6.3)	–
3 months	14 (38.9)	19 (52.7)	9 (45.0)	9 (45.0)	5 (31.2)	10 (62.5)
6 months	18 (50.0)	27 (75.0)	11 (55.0)	15 (75.0)	7 (43.8)	12 (75.0)
9 months	20 (55.5)	28 (77.8)	12 (60.0)	16 (80.0)	8 (50.0)	12 (75.0)
12 months	25 (69.4)	34 (94.4)	14 (70.0)	20 (100.0)	11 (68.8)	14 (87.5)

CNI, calcineurin inhibitor.

**Figure 3:**
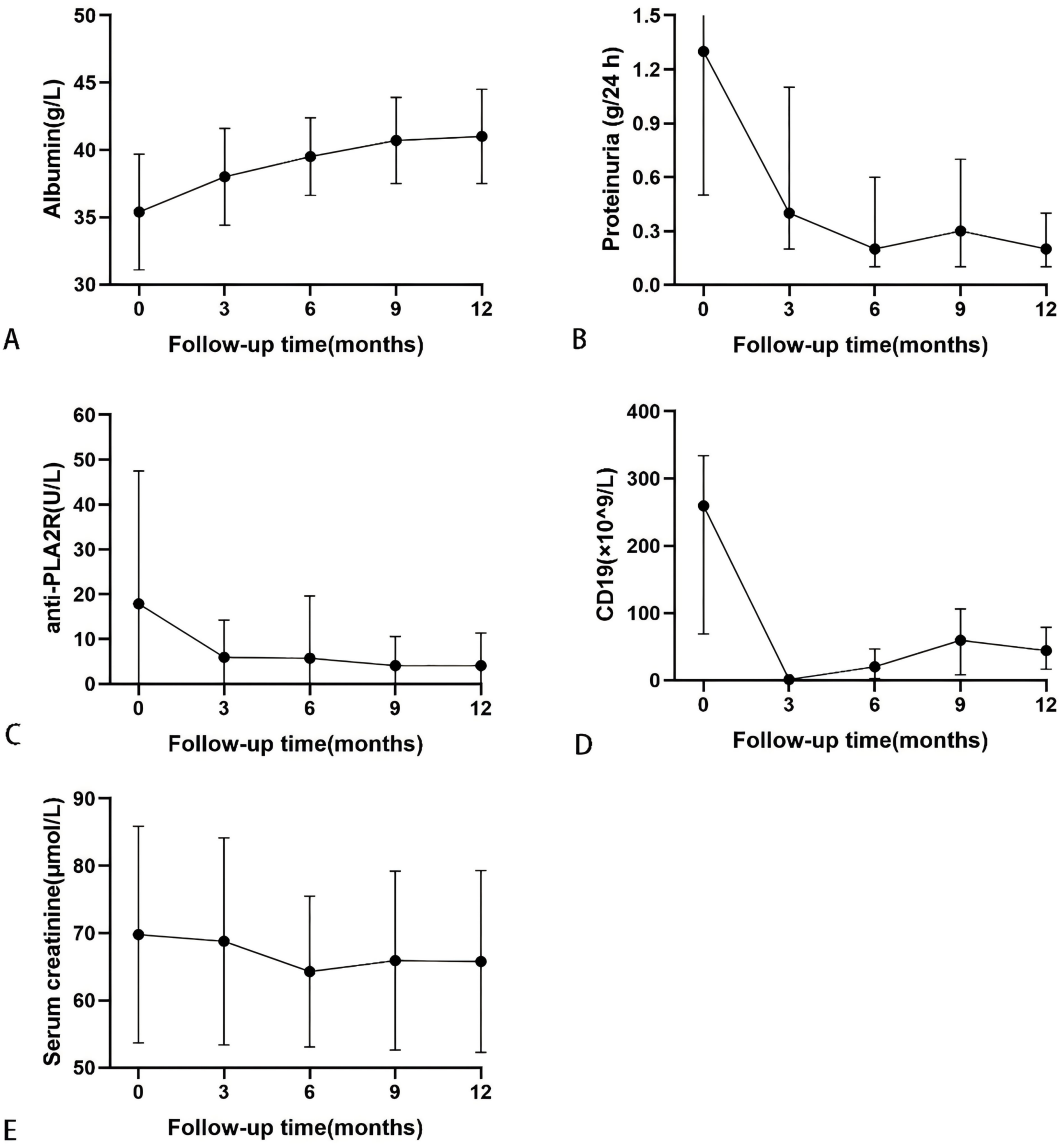
Serial levels of albumin **(A)**, proteinuria **(B)**, anti-PLA2R antibody **(C)**, the absolute values of CD19 **(D)**, and serum creatinine **(E)** after rituximab treatment in patients who had been followed up for 12 months. Data are presented as mean ± SD **(A, C, E)** or the medians (interquartile range) over time **(B, D)**.

During the follow-up, serum albumin levels demonstrated an overall upward trend, while levels of anti-PLA2R antibodies, 24-hour urinary protein, and absolute CD19+ cell numbers generally decreased, with creatinine levels remaining stable, as shown in **Figure 3**. At 12 months, serum albumin levels increased from 35.5 ± 4.3 g/L to 41.0 ± 3.5 g/L; urinary protein levels decreased from 1.3 (0.5, 2.4) g/24 h to 0.2 (0.1, 0.4) g/24 h; serum creatinine dropped from 69.8 ± 16.1 µmol/L to 65.8 ± 13.9 µmol/L; and anti-PLA2R antibody titers decreased from 4.2 (2.0, 16.4) U/mL to 2.0 (2.0, 2.0) U/mL. Among the eight patients who were initially positive for anti-PLA2R antibodies, 6 achieved a shift to negative (titer <2 U/mL). There were 32 patients with titers <4 U/mL and 31 with titers <2 U/mL, as detailed in **Table 4**.

**Table 4:** Clinical characteristics of PMN patients after rituximab treatment for 12 months.

Characteristic	Total(n=36)	Drug-dependent group (n=20)	Partial remission or immune non-remission group (n=16)	*P*
Number of withdrawals, n(%)	34 (92.5)	20 (100.0)	14 (87.5)	0.104
Time to reach withdrawal(months)	5.0 ± 4.1	5.3 ± 3.7	4.6 ± 4.5	0.597
Proteinuria(g/24 h)	0.2 (0.1, 0.4)	0.2 (0.1, 0.4)	0.3 (0.1, 0.6)	0.204
Albumin(g/L)	41.0 ± 3.5	41.8 ± 2.6	39.9 ± 4.4	0.159
Anti-PLA2R antibodies(U/mL)	2.0 (2.0, 2.0)	2.0 (2.0, 2.0)	2.0 (2.0, 2.1)	0.281
Anti-PLA2R antibody positivity, n(%)	2 (5.6)	1 (10.0)	1 (6.3)	–
Anti-PLA2R antibodies<2U/mL, n(%)	31 (85.0)	18 (90.0)	13 (81.3)	0.451
Serum creatinine(μmol/L)	65.8 ± 13.9	65.4 ± 13.8	66.3 ± 16.7	0.872
The absolute values of CD19(/μl)	44.1 (16.4,78.9)	50.6 (18.9, 83.0)	30.9 (8.6, 56.7)	0.227

Values are presented as numbers (%), medians (interquartile range), or means ± SD.

PMN, primary membranous nephropathy; PLA2R, phospholipase A2 receptor.

Anti-PLA2R positivity was defined by a value >20 U/mL.

In later stages of follow-up, three patients experienced a relapse of proteinuria, accompanied by increases in anti-PLA2R antibody titers and B-cell reconstitution. One patient relapsed at 15 months without achieving complete immunological remission by 12 months. Two patients relapsed at the 24 months; neither had received a second induction treatment at 6months. Despite discontinuing medication by the 12th month and achieving CR for proteinuria, all three patients underwent relapse. Following this, each patient received an additional 1g dose of RTX; one achieved PR after 3 months, and another after 6 months, both achieving an antibody shift to negative. The third patient did not undergo subsequent follow-up examination. Throughout the follow-up period, all patients maintained stable renal function, with no progression to ESRD.

### Safety analysis

Throughout the treatment and follow-up period, most patients tolerated RTX well. However, four patients experienced adverse reactions. Specifically, two had infusion reactions characterized by symptoms of chest tightness, breath-holding, and accelerated heart rate. These reactions were managed immediately by discontinuing the infusion and administering oxygen, low-dose steroids, and antiallergic medications, resulting in gradual symptom relief. Additionally, one patient reported pain in the limb receiving the infusion; this pain was tolerable and resolved upon cessation of the infusion. Another patient developed a pulmonary infection attributed to the novel coronavirus and required hospitalization; during this period, the patient’s proteinuria levels increased but returned to clinical remission as their condition improved. No cases of malignant tumors, lethal adverse events, opportunistic infections, or other adverse events related to ongoing immunosuppression were reported during the follow-up period.

## Discussion

The 2021 KDIGO guidelines state that CNI monotherapy is suboptimal for PMN given its limited effectiveness and association with high relapse rates following 6–12 months of treatment with rapid dose reduction. Despite this, CNIs may be considered for patients with normal eGFR and moderate progression risk, as they can shorten the duration of proteinuria and facilitate clinical remission. In cases of high progression risk, it is recommended that CNI therapy be followed by RTX therapy. However, in clinical practice, some patients require long-term CNI maintenance. Consequently, this study explores the use of RTX for patients either dependent on CNIs or those exhibiting suboptimal responses following extended (>12 months) treatment periods. We assess the efficacy of RTX in facilitating the withdrawal from immunological drug dependence. Our retrospective analysis confirms that adjunctive RTX therapy is effective in helping patients overcome CNI-dependence and improves the therapeutic outcomes while mitigating the risks associated with prolonged drug use.

Previous studies have considered alternative treatments for CNI-dependent NS patients. Mycophenolate mofetil (MMF) (24, 25), often considered for those requiring long-term CNI management, offers benefits such as lack of nephrotoxicity and fewer hematologic and gastrointestinal side effects, alongside a decreased need for GC. However, MMF does not demonstrate superior effectiveness in maintaining remission or reducing relapse rates after CNI cessation. Additionally, the combination of GC with CYC as an alternative to CNIs is limited by risks of malignancy and gonadotoxicity (26). In contrast, RTX offers a robust alternative, overcoming CNI dependency, enhancing complete remission rates, and featuring lower incidences of relapse and side effects, thus offering significant advantages.

CNI impedes T-cell activation and proliferation by inhibiting the phosphatase activity of calcineurin, thereby indirectly reducing B-cell activation and antibody production. However, this process is concentration-dependent; as CNI dosage decreases, T-cell activation and proliferation resume, prompting B-cell antibody production and recurrent proteinuria. Our study found that the majority (65%) of patients had not achieved complete immunological remission prior to receiving RTX infusion. Elevated antibody levels are indicative of potential disease relapse; thus, even if clinical remission is attained with CNI treatment, a high relapse rate is possible without immunological remission. Identifying a treatment strategy that enhances both clinical and immunological remission rates is imperative to improve prognosis.

Research indicates that following the initial course of RTX infusion, peripheral B lymphocyte counts significantly decrease, with nearly complete elimination within 1–3 months. Recovery typically begins between 6 and 8 months. Following this initial course, most anti-PLA2R antibodies gradually decline, leading to immunological remission. This effect is attributed to RTX’s specific targeting of CD20^+^ B cells, effectively eliminating them and reducing the production of anti-PLA2R antibodies. Following B-cell depletion, short-lived plasma cells and some long-lived plasma cells undergo natural apoptosis, although a small number of long-lived or permanent plasma cells may remain uncleared (27, 28). Some patients received a second course of RTX at the 6-month mark following the initial induction therapy, which further aids in the clearance of CD20^+^ B cells. Consequently, after 12 months, antibody clearance is more comprehensive, and rates of immunological remission are higher. Furthermore, the depletion of B lymphocytes diminishes the antigen-presenting process, which contributes to the gradual development of immune tolerance. Therefore, even if B cells regenerate after 6–8 months, the absence of antigen presentation prevents these cells from producing antibodies. Compared to CNI treatment, RTX therapy offers a more complete solution, independent of drug dosing, and is associated with lower relapse rates. Research by Camps et al. (29), which aligns with our findings, demonstrated that after two doses of RTX induction therapy, all 13 patients with CNI dependency achieved clinical remission, and among three who relapsed, clinical remission was maintained at 30 months post-second dose, eliminating the need for long-term medication. Therefore, RTX not only addresses CNI dependency but also enhances clinical and immunological remission rates, effectively reducing the likelihood of relapse.

In a previous study, we found (15) that extending the combination of CNI with GC from 12 to 30 months, while excluding cases of insufficient CNI dosage, did not significantly improve remission rates. The STARMEN trial (30)suggested that a sequential regimen of TAC and RTX did not provide significant benefits over an alternating regimen of GC and CYC. However, in that trial, TAC administration was reduced after only six months and only 1g of RTX was administered. Both CNI and RTX dosages were comparatively low, contributing to reduced remission rates. Based on these findings, we conducted a follow-up experiment in which a second cohort of patients, despite achieving guideline recommended CNI dosages, failed to attain CR or complete immunological remission. Nevertheless, when a sufficient dose of RTX was combined, both clinical and immunological remission rates significantly improved, validating the effectiveness of our revised treatment approach.

Two patients relapsed during the subsequent follow-up period, and they did not receive a second induction treatment after six months. These observations underscore three critical considerations for treatment: ensuring the complete clearance of anti-PLA2R antibodies to achieve full immunological remission, recommending a second course of RTX six months post-initial regimen, and emphasizing vigilant infection prevention strategies because of diminished immunity following RTX infusion. Two patients did not overcome their dependency on CNI; one owing to persistent proteinuria not reaching CR, and the other failed to achieve complete immunological remission, notably missing the second RTX course. These challenges could be associated with insufficient RTX dosage. Additionally, internalization and degradation of the CD20 antibody complex (31, 32), as well as the emergence of anti-RTX antibodies (33), might prevent complete B-cell depletion and the eradication of anti-PLA2R antibodies (34, 35). We propose the consideration of second-generation anti-CD20 monoclonal antibodies, such as obinutuzumab, for such patients (36, 37), which may assist in overcoming CNI dependency.

In conclusion, the incorporation of RTX in the treatment of patients with PMN who are dependent on long-term CNI or have only achieved partial remission, significantly increases the likelihood of achieving both complete clinical and immunological remission. Furthermore, RTX use can effectively reduce relapse rates and mitigate the risks associated with extended use of immunosuppressives. This study contributes novel insights into the efficacy of RTX for patients specifically struggling with CNI dependency or suboptimal responses to CNI therapy, thereby proposing new avenues for clinical treatment strategies. Nevertheless, the study’s limitations—its single-center retrospective design and small sample size—necessitate further validation through larger clinical trials. Additionally, more research is needed to explore alternative therapies for patients who remain CNI-dependent.

## Data Availability

The raw data supporting the conclusions of this article will be made available by the authors, without undue reservation.
